# Dietary Habits, Nutrition Intake, and Alcohol Consumption Based on Types of Smoking and Smoking Status: A Cross-Sectional Study

**DOI:** 10.3390/nu16223881

**Published:** 2024-11-14

**Authors:** Kiho Miyoshi, Yuki Kimura, Takashi Miyawaki

**Affiliations:** 1 Field of Food and Nutrition, Department of Living Environment, Graduate School of Home Economics, Kyoto Women’s University, Kyoto 605-8501, Japan; 2 Department of Food and Nutrition, Graduate School of Home Economics, Kyoto Women’s University, Kyoto 605-8501, Japan

**Keywords:** dietary intake, nutrition intake, heated tobacco product, smoker, alcohol

## Abstract

Background/objectives: Smoking increases the risk for multiple lifestyle-related diseases. In Japan, consumption of heated tobacco products (HTPs), a novel cigarette type, is rising. However, no studies have yet compared dietary habits among HTP smokers. This study assessed food and nutrient intake and alcohol consumption by smoking status (non-smokers, cigarette smokers, HTP smokers). Methods: This cross-sectional study included 237 HTP smokers, 242 cigarette smokers, and 178 non-smokers (50% each male and female). The Brief Self-Administered Diet History Questionnaire was administered as a nutritional survey, and smokers were asked about their smoking status, including smoking history and the number of puffs smoked per day. Results: A significantly higher intake of meat was seen in HTP smokers than in cigarette smokers (*p* = 0.038), and HTP smokers showed the lowest intake of vitamin D in all groups. HTP and cigarette smokers had a lower intake of certain vitamins, minerals, and dietary fiber compared with non-smokers. The rate of habitual drinkers (at least one drink a month) and their alcohol consumption (g/day) were significantly lower in non-smokers (58%, 1.3 g) than in HTP smokers (67%, 4.8 g) and cigarette smokers (70%, 7.1 g) (*p* = 0.031). Additionally, after adjusting for sex and smoking status in a multiple regression analysis, the number of puffs was a significant contributor to alcohol intake in HTP smokers (β = 0.296, *p* < 0.001). Conclusions: This study identified significant dietary, nutritional, and alcohol consumption differences based on smoking status, underscoring the need to consider both diet and smoking type in nutritional counseling and smoking cessation guidance.

## 1. Introduction

Smoking is a risk factor for the onset and exacerbation of many lifestyle-related diseases, including cancer, myocardial infarction, and metabolic syndrome. For example, smokers have significantly lower taste sensitivity than non-smokers do [1]. This decreased taste sensitivity leads to an increased use of salt, sugar, and other seasonings, in turn leading to the development of hypertension, diabetes, and other diseases. Furthermore, the dietary content and nutrient intake status of smokers reportedly differ from those of non-smokers [2]. In recent years, the use of heated tobacco products (HTPs), a new type of cigarette, has increased rapidly. In Japan, one in four smokers used HTPs, according to the National Health and Nutrition Survey conducted in 2019, and the use rate will likely continue to increase in the future.

Although there have been studies on dietary and nutrient intake in smokers, ex-smokers, and non-smokers [2,3,4,5], no studies have compared dietary intake and nutrient intake among HTP smokers. Understanding differences in eating and alcohol consumption habits by smoking status is important for registered dietitians and health educators involved in helping individuals to make healthy choices. Moreover, the combination of cigarette smoking and alcohol consumption increases adverse health outcomes such as the occurrence of throat cancer [6], but few studies have investigated the combination of HTP smoking status and alcohol consumption. Thus, this study aimed to examine differences in eating habits, food intake, nutrient intake, and alcohol consumption according to smoking status (non-smokers, cigarette smokers, and HTP smokers) to reveal nutritional problems and provide satisfactory nutritional assessment and guidance. This would enable us to build high-quality clinical nutrition for health promotion and disease prevention.

## 2. Materials and Methods

### 2.1. Participants

**Figure 1:**
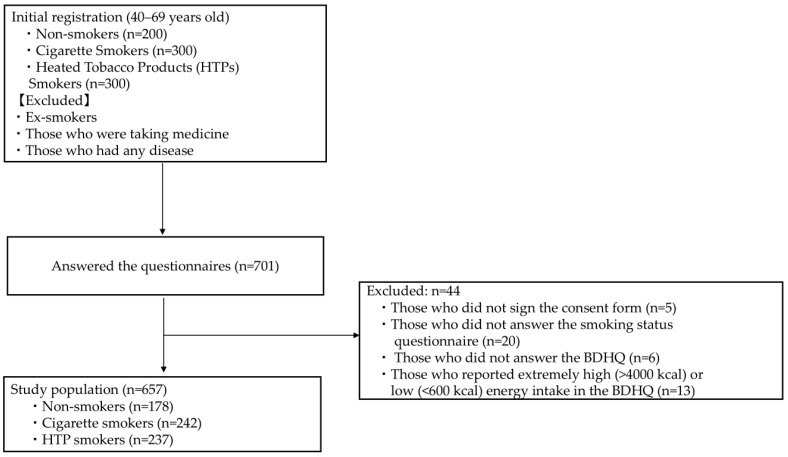
Study flow diagram of participants. BDHQ, Brief Self-Administered Diet History Questionnaire; HTP, heated tobacco product.

In total, 800 potential Japanese participants (300 cigarette smokers, 300 HTP smokers, and 200 non-smokers) were recruited and screened online using a research agency (Asmarq Corp., Tokyo, Japan). Participants were recruited from all over Japan to avoid bias in the survey area. Figure 1 shows the flow diagram of participant selection. The eligibility criteria included (a) being aged 40–69 years and (b) HTP and cigarette smokers who had been smoking for at least half a year, such that the age of each group was similar. Ex-smokers, individuals with any self-reported diseases, and those taking any medications were excluded from the study.

To mitigate selection bias, half of the participants were male individuals, and the other half were female individuals (self-reported), and they were recruited from all over Japan. The recruitment period was 2–8 March, and data were collected from 15 March to 18 April 2022. We established a catchment pool at our university and distributed questionnaires via mail to eligible participants, receiving responses from 701 individuals (response rate: 87.6%). Exclusions were made for respondents who did not sign the consent form (n = 5), did not provide information on their smoking status (n = 20) or their dietary habits (Brief Self-Administered Diet History Questionnaire [BDHQ], n = 6), or reported extremely high (>4000 kcal) or low (<600 kcal) energy intakes in the BDHQ (n = 13). The final study cohort included 178 non-smokers, 242 cigarette smokers, and 237 HTP smokers, totaling 657 participants (final response rate: 82.1%).

### 2.2. Questionnaires

The participants were asked to complete two questionnaires.

The first questionnaire collected information on the participants’ sex, age, body weight, and height. Smokers were asked about their smoking status, such as smoking history and number of puffs (cigarettes or HTP smoking) per day. The body mass index (BMI) was calculated using height and weight. The Brinkmann Index, an assessment of exposure to smoking [7] used in smoking cessation in Japan, was calculated from the smoking history and the number of puffs per day. The Tobacco Dependence Screener (TDS) [8] and Fagerström Test for Nicotine Dependence (FTND) [9] were used to assess nicotine dependence. The TDS is used for smoking cessation treatment in Japan and was developed to diagnose nicotine dependence from a psychiatric perspective in accordance with the WHO’s International Classification of Diseases, Tenth Edition (ICD-10) and the revised third and fourth editions (DSM-III-R, DSM-IV) of the American Psychiatric Association’s “Guide to the Classification and Diagnosis of Psychiatric Disorders”. It consists of 10 questions, with a score of 1 for “yes” and 0 for “no”. The total score for the 10 questions was used to determine the level of dependence. Five or more points is considered “nicotine dependence”. The FTND consists of six questions. Depending on the responses, the participants received a score of 0–3 for each question. Total scores of 0 to 2 were assessed as “low nicotine dependence”, 3 to 6 points indicated “medium dependence”, and 7 to 10 points indicated “high dependence”. The FTND is used to assess the physiological dependence on smoking.

The second questionnaire gathered information on habitual dietary/food intake using the BDHQ for non-smokers, cigarette smokers, and HTP smokers. The BDHQ is a four-page, structured, self-administered, and previously validated questionnaire that estimates the dietary intake of 58 common foods and beverages in Japan [10,11]. The BDHQ assesses dietary habits during the preceding month and consists of the following five sections: (1) intake frequency of 46 food and non-alcoholic beverage items; (2) daily intake of rice, including the type of rice (refined or unrefined) and miso soup; (3) frequency of alcoholic beverage consumption and amount per drink for five alcoholic beverages; (4) usual cooking methods; and (5) general dietary behavior. The dietary Na/K ratio, an indicator of sodium and potassium intake, was calculated from the amounts of sodium and potassium ingested. The dietary Na/K ratio is a significant risk factor for mortality from hemorrhagic stroke, cardiovascular disease, and all-cause mortality in the Japanese population [12].

### 2.3. Statistical Analysis

Data were analyzed using IBM SPSS Statistics for Windows version 28 (IBM Corp., Armonk, NY, USA). Data were tested using the Shapiro–Wilk normality test, and a normal distribution was not found. Therefore, the data have been presented as medians (first and third quartiles). Kruskal–Wallis, Bonferroni correction, and Mann–Whitney U tests (cigarette smokers vs. HTP smokers) were used to assess significant intergroup differences in the characteristics of participants and nutritional/food intake. The Fisher’s exact test was used to compare the rate of alcohol drinkers/never-drinkers. A drinker was defined as an individual who consumes alcohol at least once a month. Spearman’s rank correlation coefficient was employed to evaluate the correlation between smoking status and alcohol consumption, using a significance level of α = 0.05. A multiple regression analysis considered sex, FTND scores, number of puffs per day, and the Brinkmann Index as independent variables, with alcohol intake values as the dependent variable. Multicollinearity among the independent variables was assessed using a variance inflation factor (VIF) threshold of ≥10. Alcohol consumption is presented separately in tables for each sex, reflecting the different health impacts specified for male and female individuals [13]. Statistical significance was established at *p* < 0.05.

### 2.4. Ethical Statement

This study was approved by the Research Ethics Committee of Kyoto Women’s University (Kyoto, Japan) (approval number: 2021-23) and performed in accordance with the guidelines of the Declaration of Helsinki. Written informed consent was obtained from all the participants.

## 3. Results

### 3.1. Participant Characteristics

Approximately half of the study cohort consisted of female participants (male individuals, n = 322; female individuals, n = 335). The median age of the participants was 53 years. No significant intergroup differences in height (*p* = 0.543), body weight (*p* = 0.535), or BMI (*p* = 0.146) based on smoking status or cigarette type were observed (Table 1). Both the cigarette smokers and HTP smokers had a median smoking history of 30 years. The HTP smokers smoked significantly more puffs per day compared with cigarette smokers (*p* = 0.009). No significant differences were observed in the Brinkmann Index (*p* = 0.167), TDS scores (*p* = 0.127), and FTND scores (*p* = 0.354).

### 3.2. Food Intake, Nutritional Intake, and Alcohol Consumption by Smoking Status

#### 3.2.1. Food Intake by Smoking Status

Table 2 compares the food intake by smoking status. A significantly higher meat intake was seen among HTP smokers than among cigarette smokers (*p* = 0.038). In addition, HTP smokers showed a significantly lower intake of potatoes (*p* = 0.003), other vegetables (*p* = 0.036), fruits (*p* = 0.007), and milk and dairy products (*p* = 0.020) but a significantly higher intake of alcoholic beverages compared with non-smokers (*p* = 0.013).

A significantly lower intake of potatoes (*p* = 0.001), sugar and sweeteners (*p* < 0.001), vegetables (including green and yellow) (*p* = 0.005), mushrooms (*p* = 0.049), fruits (*p* < 0.001), seaweed (*p* = 0.004), and milk and dairy products (*p* < 0.001) but a significantly higher intake of alcoholic beverages was seen among cigarette smokers than among non-smokers (*p* < 0.001). No differences were observed among the three groups in terms of intake of cereals (*p* = 0.313), pulp (*p* = 0.092), fish and shellfish (*p* = 0.081), eggs (*p* = 0.806), non-alcoholic beverages (*p* = 0.235), seasonings (*p* = 0.404), and fats and oils (*p* = 0.105). Food intake by sex is presented in Table S1.

#### 3.2.2. Nutritional Intake by Smoking Status

Table 3 compares the nutrient intake of the participants by smoking status. There were no significant differences in energy or carbohydrate intake between HTP smokers, cigarette smokers, and non-smokers (energy: *p* = 0.246, carbohydrate: *p* = 0.633). The protein/energy ratio and fat/energy ratio were lower in both cigarette and HTP smokers than in non-smokers (cigarette smokers: protein–energy ratio: *p* < 0.001, fat–energy ratio: *p* < 0.001, HTP smokers: protein–energy ratio: *p* = 0.041, fat–energy ratio: *p* = 0.009), the median protein/energy ratio for all three groups fell within the 13–20% range, and the fat/energy ratio fell within the 20–30% range recommended by the Dietary Reference Intakes for Japanese People. Mineral intakes of calcium (*p* = 0.006), phosphorus (*p* = 0.024), zinc (*p* = 0.033), and copper (*p* = 0.046) were significantly lower in HTP smokers than in non-smokers. Cigarette smokers had a significantly lower intake of potassium compared with both non-smokers and HTP smokers (vs. non-smokers: *p* < 0.001, vs. HTP smokers: *p* = 0.043), and calcium (*p* < 0.001), magnesium (*p* = 0.007), phosphorus (*p* < 0.001), iron (*p* < 0.001), zinc (*p* < 0.001), and copper (*p* = 0.006) compared with non-smokers. Although there were no significant differences in sodium levels (*p* = 0.227), the dietary Na/K ratio was significantly higher in HTP and cigarette smokers than in non-smokers (HTP smokers: *p* = 0.023, cigarette smokers: *p* = 0.001). HTP smokers showed the lowest intake of vitamin D in all groups and was significantly lower compared with non-smokers (*p* = 0.007). HTP smokers had significantly lower intakes of B-1 (*p* = 0.018), B-2 (*p* = 0.040), B-12 (*p* = 0.047), and pantothenic acid (*p* = 0.006) than non-smokers did. Cigarette smokers had a significantly lower intake of vitamins A (*p* < 0.001), E (*p* < 0.001), K (*p* = 0.002), B-1 (*p* < 0.001), B-2 (*p* < 0.001), B-6 (*p* = 0.003), B-12 (*p* = 0.004), folic acid (*p* < 0.001), pantothenic acid (*p* < 0.001), and C (*p* < 0.001) compared with non-smokers.

Dietary fiber intake was significantly lower in both HTP and cigarette smokers than in non-smokers (HTP smokers: *p* = 0.009, cigarette smokers: *p* < 0.001).

Results by sex are shown in Table S2.

#### 3.2.3. Alcohol Consumption by Smoking Status

Table 4 compares the alcohol consumption between the groups. Both the rates of alcohol consumption and median alcohol intake (g/day) were significantly higher in HTP and cigarette smokers than in non-smokers (non-smokers: 58%, 1.3 g, cigarette smokers: 70%, 7.1 g, and HTP smokers: 67%, 4.8 g). Alcohol consumption was also significantly higher in male and female cigarette smokers. Both male and female HTP and cigarette smokers consumed more than the amount that increases the risk of lifestyle-related diseases, as defined by Health Japan 21 (male individuals: <40 g, female individuals: <20 g) [13] in the third quartile range (third quartile: male HTP smokers: 49.1, female HTP smokers: 33.9, male cigarette smokers: 54.3 g, female cigarette smokers: 50.1 g).

**Figure 2:**
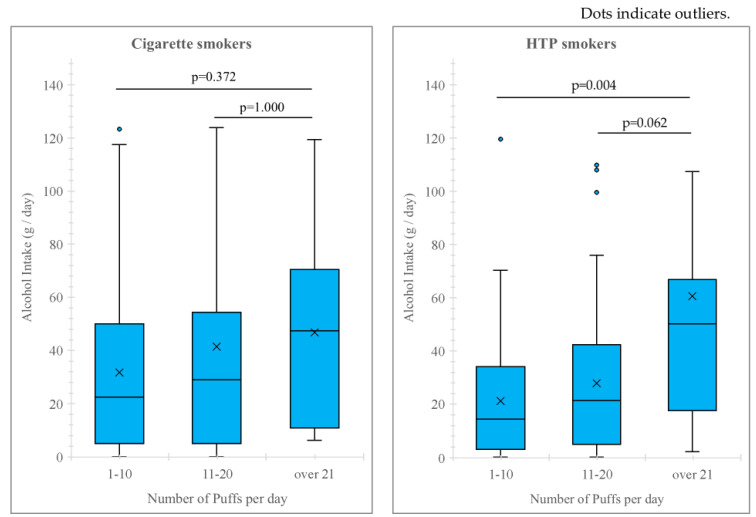
Relationship between alcohol intake and number of puffs per day. Dots indicate outliers.

In HTP smokers, there was a significant correlation between alcohol intake and the number of puffs per day and the Brinkmann Index (number of puffs per day: r = 0.269, 95% CI: 0.112–0.412, *p* < 0.001, Brinkmann Index: r = 0.270, 95% CI: 0.114–0.414, *p* < 0.001). However, alcohol consumption showed no correlations with smoking history (r = 0.152, 95% CI: −0.009–0.305, *p* = 0.057), TDS (r = −0.022, 95% CI: −0.182–0.139, *p* = 0.783) and the FTND score (r = 0.112, 95% CI: −0.050–0.268, *p* = 0.158) in HTP smokers. In cigarette smokers, alcohol consumption was significantly correlated with the number of puffs per day (r = 0.157, 95% CI: 0.001– 0.306, *p* = 0.042), Brinkmann Index (r = 0.162, 95% CI: 0.010–0.314, *p* = 0.032), and FTND scores (r = 0.159, 95% CI: 0.004–0.307, *p* = 0.039), although alcohol consumption showed no correlation between smoking history (r = 0.058, 95% CI: −0.098–0.306, *p* = 0.452), and TDS scores (r = −0.015, 95% CI: −0.170–0.140, *p* = 0.843). Multiple regression analysis, adjusted for sex, the number of puffs, Brinkmann Index, and FTND scores—which demonstrated significance in single regression analysis—revealed that the number of puffs was a significant contributor to alcohol intake in HTP smokers (β = 0.296, 95% CI: 0.65–2.01, *p* < 0.001). However, no significant contributors were identified between alcohol intake and smoking status in cigarette smokers. Among HTP smokers, participants who smoked over 21 puffs per day consumed significantly more alcohol compared with those who smoked 1–10 puffs per day, while in cigarette smokers, these two groups showed no significant differences (*p* = 0.894) (Figure 2).

## 4. Discussion

**Figure 3:**
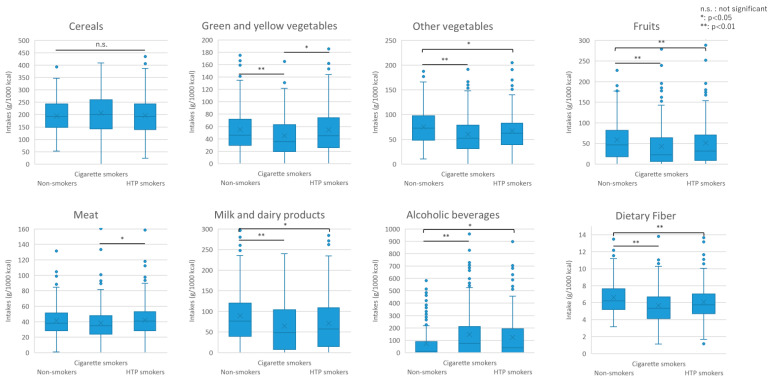
Graphical representation of food intake. Dots indicate outliers.

**Figure 4:**
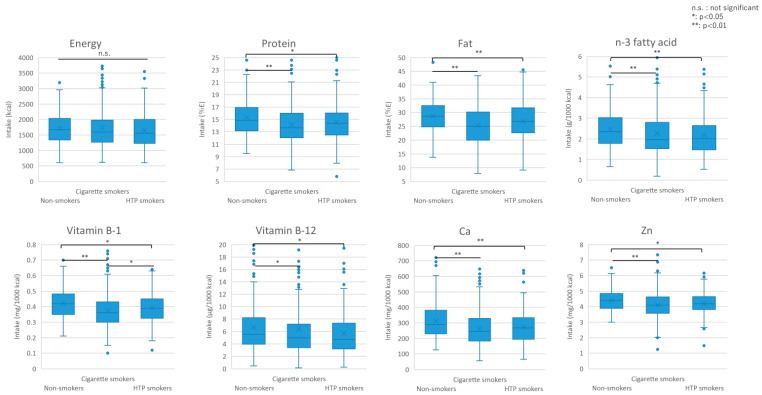
Graphical representation of nutrition intake. Dots indicate outliers.

This study is the first to investigate nutrition, food, and alcohol intake based on the types of smoking (HTP smokers, cigarette smokers, and non-smokers) and smoking status across Japan. Graphical representations of food and nutrition intakes are presented in Figure 3 and Figure 4, respectively.

We found that HTP smokers consumed significantly more meat compared with cigarette smokers. HTP and cigarette smokers had significantly lower intakes of other vegetables, potatoes, fruits, and dairy products compared with non-smokers. Regarding nutrient intake, HTP smokers showed significantly lower intakes of protein, fat, certain minerals and vitamins, and dietary fiber. In addition to nutrients, HTP and cigarette smokers had higher dietary Na/K ratios than non-smokers did. In terms of alcohol consumption, HTP and cigarette smokers had higher rates of habitual drinking and alcohol intake than non-smokers did. Furthermore, in HTP smokers, alcohol consumption showed a significant positive correlation with smoking status, including the number of puffs smoked per day.

Conventional cigarettes are smoked by burning tobacco leaves rolled in paper. In contrast, HTP cigarettes use heated-not-burnt products and produce aerosols from tobacco leaves by heating them with dedicated instruments. While HTPs reportedly produce lower concentrations of carbon monoxide than cigarettes, they contain propylene glycol and glycerol, which are present in much smaller amounts in cigarettes [14].

As for the habitual diet, HTP smokers had a significantly lower intake of potatoes, other vegetables, fruits, and dairy products than non-smokers did. Our findings are consistent with those of a previous study [3], which showed a significantly lower intake of potatoes, sweet, green, and yellow vegetables, other vegetables, fruits, and dairy products in cigarette smokers compared with non-smokers in Japan. In this nutrient intake survey, HTP smokers consumed significantly less fiber compared with non-smokers, consistent with their lower intake of potatoes, other vegetables, and fruits, which contain abundant dietary fiber. Dietary fiber increases the bulk of stools and helps prevent constipation. A previous cross-sectional study reported that cigarette smokers were more constipated than non-smokers [15]. It is possible that smokers, including HTP smokers, are more constipated due to the effects of nicotine on the autonomic nervous system, which means smoking releases the sympathetic neurotransmitter norepinephrine [16], in addition to their lower intake of fiber-rich foods. The composition of intestinal bacteria in cigarette smokers reportedly differs from that in non-smokers [17]. The underlying causes include immunosuppression, increased oxidative stress, altered intestinal barrier function, and altered acid–base equilibrium [18]. Moreover, because dietary fiber is a prebiotic that feeds the intestinal microbiota, low dietary fiber may contribute to changes in the composition of the intestinal microbiota.

Lower dietary energy density, that is, lower intake of vegetables and fruits, has been reported in cigarette smokers [19]. Consistent with these findings, in this study, HTP and cigarette smokers showed significantly lower intake of fruits, dairy products, and vegetables compared with non-smokers. Studies on the dietary and nutrient intakes of American and Canadian smokers also reported that smokers had a low intake of vegetables and fruits [19,20]. A study that investigated why smokers consume fewer fruits and vegetables compared with non-smokers showed that fruits, dairy products, and vegetables were associated with reduced craving for smoking [21]. Another study investigating foods that make cigarettes taste bad listed fruits and vegetables, caffeine-free beverages, and dairy products [22]. Because these foods make cigarettes taste bad, smokers may not like them and consume less of them. Another possible reason is that smokers may find vegetables and fruits less flavorful as their taste sensitivity is reduced due to morphological changes in the fungiform papillae on their tongues [23]. Additionally, smokers’ health-related quality of life is not as good as that of non-smokers [24]; they are less health-conscious and, therefore, consume less vegetables and fruits.

A study of nutritional intake showed that HTP and cigarette smokers had significantly lower protein–energy and fat–energy ratios compared with non-smokers. They also showed significantly lower levels of calcium, phosphorous, zinc, vitamins B-1, B-2, and B-12, and pantothenic acid compared with non-smokers. In a previous report on Japanese participants, compared with non-smokers, cigarette smokers showed lower levels of fat–energy ratio, lower intakes of β-carotene, and vitamins B-1, B-2, C, K, and Ca, which was generally consistent with the present results. However, this is the first study to clarify the nutrient intake in HTP smokers. In contrast to a previous study that found similar protein–energy ratios in cigarette smokers and non-smokers [3], we found that cigarette and HTP smokers had significantly lower protein–energy ratios compared with those in non-smokers. In the INTERMAP study conducted in Japan, China, the U.K., and the U.S., Japanese cigarette smokers had protein–energy ratios similar to those of non-smokers but significantly lower plant-based protein–energy ratios compared with those in non-smokers [2]. One possible reason for the lower protein–energy ratio in HTP smokers in this study may be that their appetite and food preferences are different from those of non-smokers. HTPs contain propylene glycol and glycerol, which are present in much smaller amounts in cigarettes [14] and have a sweet taste, which may alter food preferences. In addition, nicotine, an addictive substance found in tobacco, was found in comparable concentrations in both cigarettes and HTP smokers [25]. Nicotine also reportedly suppresses appetite [26]. In this study, HTP smokers smoked more puffs per day, which may have affected their preference for food and led to decreased intake. Compared with non-smokers, HTP and cigarette smokers showed a significantly lower intake of zinc, which affects taste; hence, the preference and intake of foods might have been different.

Proteins can be from animal or vegetable sources. In this study, HTP smokers had significantly higher intakes of meat compared with cigarette smokers. The possible reason for this is their weaker preference for plant proteins, as shown in previous studies [2]. The preference for meat may be explained by the fact that animal proteins make cigarettes taste better [22], thereby increasing the craving for smoking in HTP smokers [21]. Higher intake of saturated fatty acids, which are found abundantly in animal products such as meat [2,20], also increases the cravings for smoking. However, further research is required to determine the taste and food preferences of HTP smokers.

Intake of other nutrients, including minerals such as calcium, phosphorous, and zinc, along with vitamins B-1, B-2, B-12, and pantothenic acid, was significantly lower in HTP smokers than in non-smokers. In HTP smokers, vitamin D exhibited the lowest intake among all groups and was significantly lower than in cigarette smokers. They also had significantly lower intake of fruits and vegetables (excluding green and yellow vegetables) compared with that in non-smokers. However, there was no observed difference in the consumption of green and yellow vegetables, which are rich in antioxidant vitamins, suggesting that although the values of vitamins C and E were lower, they were not statistically significantly different. A previous study reported low intakes of β-carotene, vitamin C, calcium, and potassium, but high dietary Na/K ratios and a high energy intake from alcohol in cigarette smokers. Furthermore, another study focusing on French male cigarette smokers also indicated a low intake of vegetables and fruits, leading to reduced levels of vitamin E, vitamin C, and carotene [27]. A meta-analysis has reported low levels of dietary fiber, vitamin C, vitamin E, and β-carotene in cigarette smokers, suggesting that the low concentrations of antioxidant nutrients could be one of the mechanisms underlying smoking-related cancer and heart disease [28]. Overall, cigarette smokers were more deficient in vitamins, minerals, and other nutrients compared with HTP smokers. This may be because many HTP smokers believe that HTPs are less harmful than cigarette smoking [29] and are somewhat more health-conscious than cigarette smokers [30]. Although the health effects of HTP smoking are still unclear in some aspects, some reports showed HTPs have a negative impact on health [31,32]. Hence, it is recommended that both cigarette and HTP smokers increase their intake of fruits and vegetables and consume more vitamins and minerals, as well as quit smoking to become healthier.

In this study, we identified the relationship between alcohol consumption and smoking status. The percentage of habitual alcohol drinkers was lowest among non-smokers (58%), compared with cigarette (70%) and HTP smokers (67%). Alcohol intake in the two smoker groups was significantly higher than that of non-smokers. Both male and female smokers consumed more than the amount that increased the risk of lifestyle-related diseases as defined by Health Japan 21 (male individuals: <40 g; female individuals: <20 g) [13] in the third quartile range, which means one in four smokers may be drinking too much alcohol. Among HTP smokers, those who smoked more than 21 puffs per day consumed more alcohol compared with those who smoked 1–10 puffs per day, and the number of puffs smoked per day was a significant contributor to alcohol intake after adjusting in multiple regression analysis. There have been various previous reports on the high alcohol intake of cigarette smokers, including that nicotine-induced stress hormones may increase ethanol intake, leading to excessive alcohol intake [33]. Moreover, alcohol interacts with nicotine to stimulate the secretion of the neurotransmitter dopamine [34], called a hedonic substance, and increases smoking cravings in both men and women [35]. Furthermore, a previous large-scale study from the U.K. [36] showed that smoking rates increased with alcohol intake. In this study, HTP smokers smoked more puffs compared with cigarette smokers, and there was a correlation between alcohol intake and smoking status, including the number of puffs, which means HTP smokers have more exposure to nicotine. Studies on the combination of alcohol and cigarette smoking have reported that heavy drinkers with a smoking habit are at a significantly higher risk of dementia [37] and throat cancer [6]. Because we observed an association between alcohol consumption and HTP smoking in this study, future studies should examine the health effects of the combination of HTP and alcohol.

This study has some limitations. First, because this was a cross-sectional study, causal relationships could not be determined. Second, although the BDHQ has been validated, its accuracy is limited. Third, all participants who were HTP smokers were previously cigarette smokers; thus, future studies should include HTP users who have never smoked cigarettes to obtain a more comprehensive understanding of the impact of HTP on diet and nutrition. Additionally, the nicotine and tar levels in cigarettes and HTP were not evaluated. In future studies, the HTP brand, ingredients, nicotine content, and tar content will need to be surveyed for health risks. Fourth, dietary and nutritional intakes were analyzed by sex but were not assessed in detail according to sex, because the health impacts on HTP use by sex are unclear. Fifth, this investigation primarily included self-reported data from participants, which can be subjective and affected by recall bias. Despite these limitations, this study was able to characterize the food and nutrient intake of cigarette smokers, HTP smokers, and non-smokers in a population that did not differ in age, BMI, or other characteristics throughout Japan.

## 5. Conclusions

This study revealed that HTP smokers, cigarette smokers, and non-smokers have different dietary habits. Both HTP and cigarette smokers have a lower intake of certain vitamins, minerals, and dietary fiber and consume more alcohol compared with non-smokers. The habitual drinker’s rate and value of alcohol intake also differed between smokers and non-smokers, and smoking status, such as the number of puffs, was correlated with alcohol consumption. Therefore, when providing nutritional and smoking cessation guidance, dietary and smoking status and smoking type should be considered in addition to sex and physical status. Future studies should assess nutritional status, including biochemical indicators, and explore the impacts of HTP on human health, including long-term impacts and potential health risks.

## Figures and Tables

**Table 1:** Characteristics of participants.

	Non-Smokers	Cigarette Smokers	HTP Smokers	*p*-Value
	(n = 178)	(n = 242)	(n = 237)	
Sex (male/female)	89/89	115/127	118/119	
Age (years)	53.0 (47.8, 62.0)	54.0 (47.0, 61.0)	52.0 (47.0, 60.0)	0.358
Male	53.0 (47.5, 62.0)	54.0 (48.0, 61.5)	53.0 (47.8, 62.0)	0.892
Female	54.0 (47.0, 61.0)	53.0 (46.0, 61.0)	51.0 (46.0, 57.0)	0.155
Height (cm)	164.0 (157.0, 170.0)	164.0 (158.0, 170.0)	165.0 (158.0, 171.0)	0.543
Male	169.9 (167.0, 174.5)	171.0 (168.0, 174.8)	171.0 (168.0, 174.0)	0.505
Female	157.0 (153.0, 161.8)	158.7 (155.0, 161.0)	158.0 (156.0, 162.0)	0.188
Body weight (kg)	58.5 (51.0, 69.0)	58.0 (49.8, 67.0)	58.6 (50.0, 68.0)	0.535
Male	67.0 (60.0, 74.3)	65.0 (60.0, 72.8)	67.0 (62.9, 73.6)	0.348
Female	51.0 (46.0, 56.7)	50.4 (45.0, 55.5)	50.7 (47.0, 56.0)	0.813
BMI (kg/m^2^)	21.8 (19.8, 24.1)	21.2 (19.3, 23.4)	21.6 (19.7, 23.8)	0.146
Male	23.0 (20.8, 25.4)	22.3 (20.8, 24.2)	23.1 (21.4, 25.2)	0.094
Female	20.6 (18.9, 22.7)	20.1 (18.4, 22.0)	20.1 (18.9, 21.7)	0.336
Smoking history (years)	-	30.0 (25.0, 40.0)	30.0 (25.0, 37.3)	0.194
Male	-	33.5 (27.3, 40.0)	32.5 (27.8, 40.0)	0.758
Female	-	30.0 (23.0, 39.0)	30.0 (23.0, 34.0)	0.098
Number of puffs (Cigarette/HTP) per day	-	10 (6, 15)	13 (8, 20)	0.009 *
Male	-	15 (8, 20)	15 (10, 20)	0.426
Female	-	10 (5, 15)	12 (7, 16)	0.005 *
Brinkman index	-	340.0 (174.3, 571.5)	370.0 (219.0, 594.0)	0.167
Male	-	419.0 (271.5, 622.5)	437.5 (267.5, 621.3)	0.798
Female	-	270.0 (126.0, 460.0)	300.0 (176.3, 500)	0.125
TDS scores	-	4 (2, 7)	5 (2, 7)	0.127
Male	-	4 (2, 7)	5 (1, 7)	0.504
Female	-	4 (1, 7)	5 (2.5, 7)	0.087
FTND scores	-	4 (2, 5)	4 (2, 5)	0.323
Male	-	4 (2, 6)	4 (2, 6)	0.533
Female	-	4 (2, 5)	4 (2, 5)	0.050

Values are presented as the median (first and third quartiles). Differences between groups were analyzed using Kruskal–Wallis and Bonferroni corrections or Mann–Whitney U tests. *: *p* < 0.05. BMI, body mass index; TDS, Tobacco Dependence Screener; FTND, Fagerström Test for Nicotine Dependence; HTP, heated tobacco product.

**Table 2:** Food intake by smoking status and types of smoking.

g/1000 kcal	Non-Smokers	Cigarette Smokers	HTP Smokers	*p*-Value
	(n = 178)	(n = 242)	(n = 237)	
Cereals	192.8 (148.9, 242.8)	201.8 (143.6, 259.5)	192.8 (139.5, 243.0)	0.313
Potatoes	17.0 (8.1, 30.6)	11.7 (6.0, 22.1) **	11.8 (6.7, 21.4) ##	<0.001
Sugar and sweeteners	27.7 (17.4, 43.6)	21.0 (11.8, 34.0) **	25.4 (12.9, 41.4)	<0.001
Pulses	32.2 (20.2, 50.1)	28.3 (14.5, 44.2)	27.2 (13.9, 49.8)	0.092
Green and yellow vegetables	45.8 (29.6, 71.7)	35.5 (19.7, 62.6) **	44.9 (25.7, 74.1) +	0.002
Other vegetables	72.7(48.4, 97.9)	52.4 (31.6, 78.5) **	62.1 (39.2, 82.8) #	<0.001
Mushrooms	5.0 (2.3, 7.9)	3.2 (1.7, 7.6) *	4.0 (2.0, 7.1)	0.043
Fruits	46.4 (18.1, 81.7)	22.2 (6.7, 63.9) **	31.4 (8.7, 70.9) ##	<0.001
Seaweed	4.8 (2.0, 9.1)	3.0 (1.4, 6.5) **	3.3 (1.8, 8.1)	0.006
Fish and shellfish	30.7 (23.0, 43.7)	27.7 (19.6, 39.7)	28.6 (18.4, 41.7)	0.081
Meat	38.0 (28.3, 51.2)	34.5 (23.7, 47.9)	40.7 (28.4, 53.0) +	0.031
Eggs	20.3 (13.2, 33.8)	20.3 (12.4, 33.1)	21.1 (10.8, 35.7)	0.806
Milk and Dairy products	76.3 (39.5, 120.0)	48.2 (7.8, 104.1) **	57.6 (14.7, 108.8) #	<0.001
Alcoholic beverages	10.3 (0.0, 89.1)	76.6 (0.0, 209.6) **	39.4 (0.0, 193.9) #	<0.001
Non-alcoholic beverages	360.1 (246.5, 474.4)	353.1 (237.5, 502.7)	378.3 (265.6, 532.1)	0.235
Seasoning	11.0 (9.2, 13.6)	10.9 (7.5, 13.9)	11.0 (7.9, 13.9)	0.404
Fat and Oil	5.8 (4.5, 7.5)	5.4 (4.0, 7.2)	5.8 (4.3, 7.7)	0.105

Values are presented as the median (first and third quartiles). Differences between groups were analyzed using Kruskal–Wallis and Bonferroni corrections. *: *p* < 0.05 and **; *p* < 0.01 show non-smokers vs. cigarette smokers; # *p* < 0.05, ## *p* < 0.01, non-smokers vs. HTP smokers. +: *p* < 0.05 show cigarette smokers vs. HTP smokers.

**Table 3:** Nutritional intake by smoking status and types of smoking.

	Non-Smokers	Cigarette Smokers	HTP Smokers	*p*-Value
	(n = 178)	(n = 242)	(n = 237)	
Energy (kcal/day)	1672.7 (1340.6, 2034.7)	1597.6 (1271.8, 1970.5)	1556.8 (1228.3, 2003.7)	0.246
Protein (%E)	14.8 (13.2, 16.9)	13.6 (12.0, 16.0) **	14.4 (12.5, 16.1) #	<0.001
Fat (%E)	28.7 (24.9, 32.5)	25.0 (20.0, 30.2) **	26.8 (22.7, 31.8) ##	<0.001
Carbohydrate (g/1000 kcal)	128.8 (112.4, 141.5)	125.1 (104.9, 142.9)	125.0 (110.8, 142.1)	0.633
Na (mg/1000 kcal)	2268.3 (1984.3, 2567.5)	2196.0 (1855.4, 2579.6)	2260.3 (1974.2, 2607.8)	0.227
K (mg/1000 kcal)	1372.3 (1135.1, 1632.9)	1178.1 (963.1, 1483.7) **	1299.0, (1068.2, 1538.4) +	<0.001
Ca (mg/1000 kcal)	290.8 (229.4, 381.9)	245.4 (183.8, 328.2) **	267.2 (194.8, 332.9) ##	<0.001
Mg (mg/1000 kcal)	135.0 (117.8, 160.2)	127.5 (108.4, 151.4) **	131.1 (115.1, 152.9)	0.01
P (mg/1000 kcal)	563.7 (485.3, 647.6)	514 (439.2, 620.4) **	532.4 (464.5, 610.0) #	<0.001
Fe (mg/1000 kcal)	4.3 (3.6, 5.0)	3.8 (3.1, 4.8) **	4.0 (3.4, 4.8)	<0.001
Zn (mg/1000 kcal)	4.4 (3.9, 4.9)	4.1 (3.6, 4.6) **	4.2 (3.8, 4.6) #	<0.001
Cu (mg/1000 kcal)	0.6 (0.5, 0.7)	0.6 (0.5, 0.6) **	0.6 (0.5, 0.6) #	0.006
Mn (mg/1000 kcal)	1.6 (1.3, 2.1)	1.5 (1.3, 2.0)	1.6 (1.3, 2.0)	0.527
Vit.A (μgRAE/1000 kcal)	368.5 (272.4, 533.4)	281.1 (202.3, 470.9) **	346.2 (228.0, 506.3) +	<0.001
Vit.D (μg/1000 kcal)	5.5 (4.0, 8.2)	4.9 (3.4, 7.1)	4.7 (3.2, 7.3) ##	0.008
Vit.E (mg/1000 kcal)	4.1 (3.3, 4.9)	3.4 (2.7, 4.3) **	3.8 (3.1, 4.7) ++	<0.001
Vit.K (μg/1000 kcal)	167.0 (113.4, 224.0)	136.8 (89.4, 200.2) **	148.6 (103.3, 210.9)	0.003
Vit.B-1 (mg/1000 kcal)	0.42 (0.35, 0.48)	0.36 (0.30, 0.43) **	0.39 (0.33, 0.45) #+	<0.001
Vit.B-2 (mg/1000 kcal)	0.75 (0.62, 0.88)	0.66 (0.54, 0.84) **	0.69 (0.57, 0.85) #	<0.001
Niacin (mg/1000 kcal)	9.4 (8.0, 10.9)	9.2 (7.4, 11.1)	9.5 (8.2, 11.1)	0.295
Vit.B-6 (mg/1000 kcal)	0.67 (0.57, 0.79)	0.60 (0.48, 0.74) **	0.63 (0.54, 0.74)	0.004
Vit.B-12 (μg/1000 kcal)	4.2 (3.4, 5.6)	3.7 (2.7, 5.4) *	3.8 (2.8, 5.4) #	0.023
Folic acid (μg/1000 kcal)	178.4 (144.1, 224.4)	156.0 (116.0, 207.9) **	169.4 (132.4, 219.0) +	<0.001
Pantothenic acid (mg/1000 kcal)	3.5 (3.1, 4.1)	3.2 (2.7, 3.8) **	3.3 (2.9, 3.9) ##	<0.001
Vit.C (mg/1000 kcal)	56.8 (40.2, 74.2)	43.3 (28.8, 63.8) **	47.9 (35.5, 72.7) +	<0.001
n-3 fatty acid (g/1000 kcal)	2.4 (1.8, 3.0)	2.0 (1.5, 2.8) **	2.0 (1.5, 2.6) ##	0.001
Dietary Fiber (g/1000 kcal)	6.2 (5.2, 7.6)	5.3 (4.2, 6.7) **	5.8 (4.7, 7.0) ##	<0.001
Salt equivalent (g/1000 kcal)	5.7 (5.0, 6.5)	5.5 (4.7, 6.5)	5.7 (5.0, 6.6)	0.223
Dietary Na/K ratio	1.6 (1.4, 1.9)	1.8 (1.5, 2.1) **	1.8 (1.5, 2.1) #	0.001

Values are presented as the median (first and third quartiles). The Na/K ratio was calculated using dietary sodium and potassium. Differences between groups were analyzed using Kruskal–Wallis and Bonferroni corrections. *: *p* < 0.05 and **: *p* < 0.01 show non-smokers vs. cigarette smokers. # *p* < 0.05, ## *p* < 0.01, non-smokers vs. HTPs smokers. +: *p* < 0.05; ++: *p* < 0.01 show cigarette smokers vs. HTP smokers. Protein and fat intakes are shown by energy ratio. Vit.: vitamin. n-3 fatty acid: omega-3 fatty acid.

**Table 4:** Alcohol consumption by types of smoking.

	Non-Smokers	Cigarette Smokers	HTP Smokers	*p*-Value
	(n = 178)	(n = 242)	(n = 237)	
Alcohol drinker/Non-drinker (n, %)	103, 58%/75, 42%	169, 70%/72, 30%	157, 67%/79, 33%	0.031
Alcohol intake (g/day)	1.3 (0.0, 15.1)	7.1 (0.0, 42.6) **	4.8 (0.0, 32.1) ##	<0.001
Male drinkers’ alcohol intake (g/day)	17.6 (2.7, 38.0)	35.8 (10.0, 54.3) **	25.5 (7.0, 49.1)	0.004
Female drinkers’ alcohol intake (g/day)	4.5 (2.0, 18.0)	13.0 (2.9, 50.1) *	16.7 (3.3, 33.9)	0.028

Values are presented as the median (first and third quartiles). Differences between groups were analyzed using Fisher’s exact test. Differences between groups were analyzed using Kruskal–Wallis and Bonferroni corrections. *: *p* < 0.05 and **: *p* < 0.01 show non-smoker vs. cigarette smokers. ## *p* < 0.01, non-smokers vs. HTP smokers.

## Data Availability

The original contributions presented in the study are included in the article/Supplementary Materials, further inquiries can be directed to the corresponding author.

## Supplementary Materials

The following supporting information can be downloaded at: https://www.mdpi.com/article/10.3390/nu16223881/s1, Table S1: Food intake by smoking status and smoking type according to sex; Table S2: Nutritional intake by smoking status and smoking type by sex.
