# Supplementary material for: Dietary Habits, Nutrition Intake, and Alcohol Consumption Based on Types of Smoking and Smoking Status: A Cross-Sectional Study
Source: Nutrients. 2024 Nov 14;16(22):3881. doi: 10.3390/nu16223881 (PMC11597212; doi:10.3390/nu16223881)
Supplement: Supplementary file 1 [file nutrients-16-03881-s001.zip › nutrients-3309187-supplementary.pdf]

Table S1. Food intake by smoking status and smoking type according to sex

g/1000 kcal		Non-smokers (n=178)	Cigarette smokers (n=242)	HTP smokers (n=237)	p-value
Cereals	Male	202.2 (159.3, 244.5)	227.4 (177.4, 286.0)	202.9 (157.3, 255.1)	0.048
	Female	182.2 (135.0, 222.0)	175.2 (131.5, 243.2)	183.2 (131.9, 224.6)	0.980
Potatoes	Male	13.6 (6.9, 28.3)	10.5 (5.9, 19.1)	11.1 (6.1, 21.5)	0.300
	Female	23.3 (11.7, 32.4)	12.7 (6.1, 24.0) **	13.3 (7.1, 21.3) ##	<0.001
Sugar and sweeteners	Male	24.5 (15.3, 35.3)	18.3 (10.2, 29.3) *	23.1 (10.1, 34.9)	0.040
	Female	32.5 (19.4, 48.9)	22.7 (12.9, 38.6) **	28.0 (16.3, 43.7)	0.006
Pulses	Male	28.9 (17.3, 41.3)	23.2 (11.5, 36.8)	26.8 (13.6, 47.1)	0.099
	Female	35.4 (22.4, 60.7)	34.0 (18.9, 51.5)	27.8 (14.6, 53.3)	0.119
Green and yellow vegetables	Male	43.7 (27.4, 68.3)	28.6 (16.0, 54.0) **	45.9 (25.8, 70.1) ++	<0.001
	Female	50.3 (32.3, 75.5)	44.6 (23.5, 71.1)	42.2 (25.5, 77.6)	0.256
Other vegetables	Male	62.7 (38.0, 80.6)	45.5 (27.4, 68.1) **	60.8 (37.8, 81.9) ++	0.001
	Female	80.4 (58.5, 110.2)	62.7 (38.1, 90.2) **	63.0 (41.6, 83.5) ##	<0.001
Mushrooms	Male	4.0 (2.1, 6.3)	2.3 (1.3, 4.1) **	2.8 (1.8, 6.0) +	0.001
	Female	6.1 (2.6, 10.5)	5.9 (2.5, 10.1)	4.6 (2.1, 8.2)	0.183
Fruits	Male	44.8 (18.4, 79.6)	19.8 (5.0, 53.9) **	28.1 (6.3, 64.3)	0.001
	Female	49.4 (17.9, 91.7)	25.4 (8.3, 72.8) *	37.3 (11.1, 74.9)	0.037
Seaweed	Male	3.0 (1.5, 7.2)	2.4 (1.3, 5.6) **	3.2 (1.5, 7.1)	0.338
	Female	7.1 (3.1, 10.7)	3.4 (1.4, 8.8) **	3.7 (2.1, 9.0) #	0.001
Fish and shellfish	Male	30.5 (23.1, 40.9)	24.1 (17.9, 31.1)	28.2 (18.1, 39.0)	0.001
	Female	32.3 (23.3, 47.6)	32.8 (21.9, 48.2)	29.1 (27.0, 53.5)	0.258
Meat	Male	36.6 (28.3, 47.3)	31.8 (22.4, 51.3)	41.7 (28.7, 51.3) ++	0.010
	Female	38.9 (28.8, 53.9)	38.1 (25.7, 49.4)	39.1 (27.0, 53.5)	0.619
Eggs	Male	17.6 (10.6, 29.6)	20.0 (11.9, 32.5)	20.2 (12.4, 37.9)	0.387
	Female	22.0 (15.3, 35.5)	20.3 (13.5, 33.6)	21.6 (10.1, 33.7)	0.318
Milk and Dairy products	Male	72.5 (39.1, 111.2)	27.1 (4.6, 91.4) **	38.0 (12.4, 95.1) ##	<0.001
	Female	81.2 (47.8, 127.8)	68.3 (14.4, 109.8)	80.1 (21.6, 121.4)	0.141
Alcoholic beverages	Male	34.2 (0.0, 167.6)	83.3 (0.0, 239.5)	61.1 (0.0, 222.9)	0.286
	Female	0.0 (0.0, 42.4)	43.8 (0.0, 196.3) **	23.4 (0.0, 166.0) #	<0.001
Non-alcoholic beverages	Male	332.5 (250.0, 432.6)	334.1 (222.4, 488.4)	377.8 (255.7, 529.7)	0.239
	Female	383.2 (245.7, 487.1)	369.8 (259.5, 513.6)	378.3 (274.7, 518.6)	0.716
Seasoning	Male	10.6 (8.9, 12.7)	11.0 (7.7, 13.7)	10.9 (8.0, 13.2)	0.954
	Female	11.6 (9.4, 14.9)	10.7 (7.5, 14.0)	11.4 (7.8, 14.4)	0.156
Fat and Oil	Male	6.0 (4.7, 7.5)	5.3 (4.2, 6.6) *	5.6 (4.3, 7.4)	0.049
	Female	5.7 (4.5, 7.5)	5.6 (3.9, 7.7)	5.8 (4.2, 8.1)	0.810

Values are presented as the median (first and third quartiles). Differences between groups were analyzed using Kruskal–Wallis and Bonferroni corrections.

*: p<0.05 and **; p<0.01 show non-smokers vs. cigarette smokers; # p<0.05, ## p<0.01, non-smokers vs. HTP smokers.

+: *p*<0.05; ++: *p*<0.01 show cigarette smokers vs. HTP smokers.

Table S2. Nutritional intake by smoking status and smoking type by sex

		Non-smokers (n=178)	Cigarette smokers (n=242)	HTPs smokers (n=237)	p-value
Energy (kcal/day)	Male	1781.1 (1401.6, 2157.9)	1736.4 (1439.2, 2343.0)	1720.9 (1405.2, 2098.8)	0.632
	Female	1574.7 (1284.1, 1849.4)	1592.6 (1207.7, 1823.1)	1387.8 (1071.2, 1751.1)	0.077
Protein (%E)	Male	14.1 (12.8, 16.1)	12.8 (11.5, 14.3) **	14.2 (12.3, 15.6) ++	<0.001
	Female	15.5 (13.8, 17.4)	15.0 (12.8, 17.4)	14.6 (12.8, 16.8) #	0.045
Fat (%E)	Male	28.2 (23.7, 31.6)	23.3 (19.7, 27.4) **	25.1 (22.2, 29.3) +	<0.001
	Female	29.1 (26.2, 33.6)	27.3 (21.5, 32.8) **	28.2 (23.4, 33.2)	0.012
Carbohydrate (g/1000 kcal)	Male	129.6 (118.4, 141.2)	129.1 (114.8, 146.1)	125.3 (109.3, 142.4)	0.466
	Female	127.7 (111.1, 141.4)	123.6 (100.7, 139.2)	124.6 (114.4, 141.5)	0.137
Na (mg/1000 kcal)	Male	2212.2 (1960.6, 2429.0)	2107.9 (1777.3, 2457.5)	2260.3 (2024.4, 2602.4) +	0.019
	Female	2381.4 (2009.1, 2690.7)	2294.7 (1916.3, 2695.5)	2266.6 (1931.4, 2672.6)	0.661
K (mg/1000 kcal)	Male	1262.5 (1092.5, 1505.6)	1091.4 (896.0, 1261.9) **	1192.9 (1034.6, 1452.7) ++	<0.001
	Female	1465.4 (1270.5, 1735.5)	1355.5 (1066.6, 1655.6)	1382.1 (1081.0, 1617.7)	0.072
Ca (mg/1000 kcal)	Male	273.7 (205.3, 340.7)	213.3 (142.3, 276.1) **	239.2 (178.8, 305.2)	<0.001
	Female	314.4 (248.6, 394.7)	289.7 (213.3, 379.5)	285.9 (217.4, 350.7)	0.076
Mg (mg/1000 kcal)	Male	131.4 (116.2, 143.9)	117.0 (104.0, 131.6) **	129.3 (111.8, 143.1) ++	<0.001
	Female	147.6 (125.3, 166.9)	136.2 (119.7, 164.6)	135.2 (118.9, 158.0	0.325
P (mg/1000 kcal)	Male	535.8 (475.4, 606.1)	475.9 (411.3, 535.8) **	514.5 (446.0, 577.6) +	<0.001
	Female	593.5 (508.5, 665.2)	566.4 (485.2, 648.2)	553.2 (487.2, 553.2)	0.127
Fe (mg/1000 kcal)	Male	4.0 (3.2, 4.7)	3.4 (2.8, 3.9) **	3.8 (3.4, 4.6) ++	<0.001
	Female	4.6 (4.0, 5.4)	4.3 (3.4, 5.1)	4.1 (3.4, 5.0) #	0.024
Zn (mg/1000 kcal)	Male	4.2 (3.8, 4.6)	3.9 (3.5, 4.2) **	4.1 (3.7, 4.6) +	0.001
	Female	4.6 (4.1, 5.0)	4.3 (3.9, 4.7) *	4.3 (3.8, 4.7) #	0.012
Cu (mg/1000 kcal)	Male	0.6 (0.5, 0.6)	0.5 (0.5, 0.6) *	0.6 (0.5, 0.6)	0.030
	Female	0.6 (0.6, 0.7)	0.6 (0.5, 0.7)	0.6 (0.5, 0.7) #	0.040
Mn (mg/1000 kcal)	Male	1.5 (1.2, 1.8)	1.5 (1.2, 1.9)	1.6 (1.3, 2.0)	0.263
	Female	1.7 (1.4, 2.1)	1.6 (1.3, 2.1)	1.5 (1.3, 2.0)	0.232
Vit.A (μgRAE/1000 kcal)	Male	338.0 (263.1, 483.0)	250.6 (160.5, 380.9) **	343.0 (223.1, 498.6) ++	<0.001
	Female	378.8 (295.5, 565.1)	335.4 (224.8, 523.8)	351.4 (236.1, 536.2)	0.048
Vit.D (μg/1000 kcal)	Male	5.4 (3.9, 7.0)	4.2 (3.0, 5.7) **	4.5 (3.1, 6.5)	0.007
	Female	5.9 (4.2, 9.1)	6.2 (4.1, 8.7)	4.8 (3.3, 8.1)	0.031
Vit.E (mg/1000 kcal)	Male	3.9 (3.0, 4.6)	3.1 (2.5, 3.8) **	3.6 (3.0, 4.3) ++	<0.001
	Female	4.3 (3.6, 5.0)	3.7 (3.0, 4.7) **	4.1 (3.2, 4.8)	0.011
Vit.K (μg/1000 kcal)	Male	151.9 (109.1, 196.7)	118.8 (79.5, 164.2) **	144.3 (111.7, 190.5) +	0.002
	Female	181.0 (129.1, 243.7)	156.8 (106.0, 233.4)	155.9 (103.3, 230.2)	0.096
Vit.B-1 (mg/1000 kcal)	Male	0.38 (0.33, 0.45)	0.33 (0.27, 0.40) **	0.38 (0.32, 0.43) ++	<0.001
	Female	0.44 (0.38, 0.51)	0.40 (0.32, 0.48) **	0.41 (0.33, 0.48) #	0.002
Vit.B-2 (mg/1000 kcal)	Male	0.70 (0.59, 0.83)	0.59 (0.47, 0.71) **	0.65 (0.54, 0.78) +	<0.001
	Female	0.79 (0.69, 0.92)	0.74 (0.62, 0.92)	0.72 (0.61, 0.90)	0.176
Niacin (mg/1000 kcal)	Male	9.1 (7.9, 10.3)	8.0 (7.0, 10.0) *	9.2 (8.0, 10.8) ++	0.002
	Female	10.1 (8.3, 11.5)	10.3 (8.1, 12.2)	9.8 (8.5, 11.4)	0.553
Vit.B-6 (mg/1000 kcal)	Male	0.62 (0.55, 0.71)	0.53 (0.22, 0.63) **	0.62 (0.54, 0.68) ++	<0.001
	Female	0.70 (0.59, 0.82)	0.68 (0.55, 0.80)	0.66 (0.55, 0.78)	0.250

Vit.B-12 (μg/1000 kcal)	Male	4.1 (3.4, 5.0)	3.2 (2.4, 4.2) **	3.8 (2.7, 5.1)	<0.001
	Female	4.4 (3.4, 6.0)	4.3 (3.3, 6.6)	3.8 (2.9, 6.0)	0.155
Folic acid (μg/1000 kcal)	Male	166.8 (133.1, 209.0)	134.5 (105.8 (175.0) **	163.0 (129.1, 207.3) ++	<0.001
	Female	194.7 (163.0, 242.3)	172.9 (131.2, 224.5)	177.0 (136.2, 226.8)	0.032
Pantothenic acid (mg/1000 kcal)	Male	3.4 (3.0, 3.9)	3.0 (2.5, 3.4) **	3.2 (2.8, 3.6) +	<0.001
	Female	3.8 (3.3, 4.2)	3.5 (2.9, 4.0) *	3.5 (2.9, 4.1)	0.031
Vit.C (mg/1000 kcal)	Male	51.9 (35.2, 70.1)	37.1 (25.3, 52.8) **	46.2 (34.9, 66.5) ++	<0.001
	Female	60.5 (46.1, 77.7)	48.9 (34.7, 73.4) *	51.6 (36.8, 74.9)	0.014
n-3 fatty acid (g/1000kcal)	Male	2.5 (1.8, 3.0)	2.1 (1.6, 2.8)	2.2 (1.7, 2.7)	0.096
	Female	2.2 (1.8, 3.0)	1.9 (1.5, 2.9)	1.9 (1.3, 2.6) ##	0.003
Dietary Fiber (g/1000 kcal)	Male	5.7 (4.9, 6.9)	5.0 (3.7, 6.3) **	5.5 (4.7, 6.7) ++	<0.001
	Female	7.0 (5.7, 8.4)	5.8 (4.5, 7.4) **	6.0 (4.9, 7.3) ##	<0.001
Salt equivalent (g/1000 kcal)	Male	5.6 (5.0, 6.1)	5.3 (4.5, 6.2)	5.7 (5.1, 6.6) +	0.019
	Female	6.0 (5.1, 6.8)	5.8 (4.9, 6.8)	5.7 (4.9, 6.7)	0.658
Dietary Na/K ratio	Male	1.7 (1.4, 2.0)	1.9 (1.6, 2.3) **	1.9 (1.6, 2.2) #	0.003
	Female	1.6 (1.4, 1.9)	1.7 (1.4, 2.1)	1.7 (1.4, 2.0)	0.178

Values are presented as the median (first and third quartiles). The Na/K ratio was calculated using dietary sodium and potassium. Differences between groups were analyzed using Kruskal–Wallis and Bonferroni corrections.
*: p<0.05 and **: p<0.01 show non-smokers vs. cigarette smokers. # p<0.05, ## p<0.01, non-smokers vs. HTPs smokers.
+: p<0.05; ++: p<0.01 show cigarette smokers vs. HTP smokers.
Protein and fat intakes are shown by energy ratio.
Vit.: Vitamin
n-3 fatty acid: omega-3 fatty acid
